# Seasonal pattern of preterm birth in the Netherlands: A population-based retrospective cohort study

**DOI:** 10.1371/journal.pone.0324873

**Published:** 2025-05-27

**Authors:** Nadine D. de Klerk, Ivar R. de Vries, Phebe B. Q. Berben, Annemarie F. Fransen, Myrthe van der Ven, Rik Vullings, M. Beatrijs van der Hout-van der Jagt, S. Guid Oei, Judith O. E. H. van Laar

**Affiliations:** 1 Department of Gynaecology and Obstetrics, Máxima Medical Center, Veldhoven, The Netherlands; 2 Department of Electrical Engineering, Eindhoven University of Technology, Eindhoven, The Netherlands; 3 Eindhoven MedTech Innovation Center, Eindhoven, The Netherlands; 4 Department of Biomedical Engineering, Eindhoven University of Technology, Eindhoven, The Netherlands; Adam Mickiewicz University Faculty of Biology: Uniwersytet im Adama Mickiewicza w Poznaniu Wydzial Biologii, POLAND

## Abstract

**Background:**

Prior research has demonstrated the impact of environmental influences, including seasonal patterns, as determinants of preterm birth, although relationships vary. This study aims to determine whether there is an association between month of conception and preterm birth or gestational age at birth in the Netherlands.

**Methods:**

This was a population-based retrospective cohort study with data collected between 2015–2019. Data originated from the national perinatal registry in The Netherlands, of which 828,574 deliveries were included.

Relationships between month of conception and birth with gestational age at delivery were investigated. Furthermore, trends in the preterm birth rate among women diagnosed with imminent preterm birth were investigated. Additionally, the relationship between outdoor temperature, humidity, hours of sunlight with preterm birth was examined to investigate their possible role in preterm birth’s pathophysiology.

**Results:**

The distribution of gestational age at birth showed significant variation over the months of conception when all births, spontaneous onset of delivery, or iatrogenic onset of delivery were considered (all p < 0.001). Preterm and term births were more common for conception between January and June, whereas birth >41 weeks of gestation was most common for conception between July and December. Seasonal or monthly variation in preterm birth rate among women diagnosed with imminent preterm birth was not significant (p = 0.103). Higher humidity was associated with higher preterm birth rates (HR = 1.003, 95% CI: 1.001–1.005), whereas higher outdoor temperature (HR = 0.998, 95% CI: 0.996–1.000) and more sunlight exposure (HR = 0.994, 95% CI: 0.995–0.998) were associated with lower preterm birth rates.

**Conclusions:**

Pregnancies conceived in January through June are associated with a higher risk of preterm birth. These outcomes demonstrate a seasonal pattern and identify environmental risk factors for preterm birth. These patterns might be a result of fluctuations in melatonin levels, governed by variation in the hours of daily sunshine over the year in the Netherlands.

## Introduction

Preterm birth (PTB), defined as birth occurring before 37 + 0 weeks of gestation, is one of the main causes of mortality in the neonatal period and infancy, as well as morbidity later in life [1–3] The burden of PTB on global public health is significant, resulting in high expenses along with negative effects on quality of life of surviving infants [4]. Over the past decade, there has not been any measurable change in the worldwide rate of PTB, despite improvements in health care systems [4]. This may be due to an incomplete understanding of PTB’s pathophysiology, and insight into modifiable risk factors could therefore be of importance [5].

Important risk factors encompass a complex combination of maternal characteristics, such as age, ethnicity, genetics, infections, and socioeconomic and lifestyle factors [6,7]. Furthermore, research has demonstrated the impact of environmental influences, including seasonal patterns, as determinants of adverse birth outcomes [8,9]. Seasonal patterns might arise from the influence of climatic variables on PTB rates [10]. Proliferation of the myometrium occurs as a result of oxytocin-induced uterine activity [10], which in turn, is enhanced in the presence of melatonin [11] and inhibited by progesterone [12]. Melatonin does so by binding to receptors, which are more pronounced in utero during labour in comparison to the antepartum period [13]. Melatonin follows a circadian rhythm with a nocturnal rise, contributing to increased night-time labour onset and delivery [13]. Both the maternal pineal gland and the placenta produce melatonin, and serum concentration increases during pregnancy [13,14]. Melatonin is highly effective in reducing oxidative stress, even more than other well-known anti-oxidants [13]. As melatonin can cross all morphophysiological barriers, it protects mother, placenta and fetus [13,15]. Based on the synergetic effect of melatonin and oxytocin, it would be interesting to investigate whether pregnancies in the range 24–37 weeks of gestation during the local fall and winter months are at higher risk of developing PTB than those in that range during the spring and summer months. Since countries at higher latitudes show large differences in seasonal hours of sunshine, and melatonin secretion correlates to ambient light intensity [16], we hypothesize that these countries might show a seasonal effect of PTB. Similar to light, temperature perception might also influence PTB rates since progesterone plays a role in thermoregulation. As higher temperatures are associated with lower progesterone levels, outdoor temperature and humidity, both influencing temperature perception, could be risk factors for PTB as well [17].

However, research regarding seasonal patterns of PTB, have shown varying findings even in countries with similar climates [18–26]. Studies investigating the relation between PTB and conception month, rather than birth month, have found results varying from one annual peak in PTB for pregnancies conceived in May/June in Japan[25], while in the US and China one annual nadir in respectively September and July, and one peak in respectively March and December/January were found [27,28]. These variations in outcomes were suggested to be related to multiple factors, amongst which climatic variables like length of exposure to light, humidity and outdoor temperature [29,30]. A significant positive correlation between temperature and the risk of PTB was found in China [31] and Australia [32].

Since seasonal variations in sunshine hours, temperature and humidity are dependent on a country’s climate and latitude, seasonal effects of PTB from research performed in different climates or latitudes cannot simply be generalized to other countries.

Furthermore, current literature mainly concerns research conducted in countries with multiple climates or large variation in latitude, or considers time of birth rather than time of conception, which complicates the interpretation of their results. The Netherlands, with its relatively high latitude (i.e., direct sunshine varying between 0 and 16 hours per day [33]) and uniform maritime climate, serves as a suitable country to further investigate the effects of climatic factors on PTB. Since no such studies have been performed in the Netherlands or surrounding countries (outside the UK [23]) yet, this research evaluates a seasonal birth pattern within the Netherlands to broaden comprehension of the risk factors linked to PTB.

## Materials and methods

### Data sources

We conducted a population-based retrospective cohort study involving women aged 13–59 years who received obstetrical care and gave birth above 23 + 6 weeks of gestation in the Netherlands between 2015 and 2019. Data from the COVID-19 pandemic was excluded as the impact of COVID-19 on pregnant women and their fetuses is poorly understood [34]. We excluded women whose delivery date was uncertain. Data originated from the anonymized national perinatal registry PERINED (https://www.perined.nl, data request 24.14). Obstetricians, neonatologists, and midwife practices in the Netherlands are requested to participate in the registration of data in the PERINED registry, covering 96–98% of all deliveries in the Netherlands [35]. Due to ethical and legal restrictions, these data are not publicly available. Access to Perined data is subject to approval and requires a formal data request. The authors conducted on-site analysis at Perined on 18 June 2024, and only aggregated, anonymized data were used in this publication. The authors do not have off-site access to the raw data and therefore are not able to share data. Interested researchers can apply for access via the Perined website (www.perined.nl). The Medical Ethics Review Committee of Máxima Medical Center has reviewed this study (N25.005) and concluded that it does not fall under the scope of the Dutch Medical Research Involving Human Subjects Act (WMO). Informed consent regarding data collection by PERINED was gathered by direct caregivers of the women.

### Outcomes and definitions

The relationship between month of conception and birth, and gestational age (GA) at delivery (subdivided in GA of 24–28 weeks, 28–32 weeks, 32–37 weeks, 37–41 weeks and >41 weeks) was investigated for both spontaneous and iatrogenic onset of birth. Furthermore, the preterm birth rate among women diagnosed with imminent preterm birth was examined in relation to conception and birth month. Lastly, the relationship between outdoor temperature, humidity, hours of sunlight and PTB, was investigated for all singleton pregnancies with a spontaneous onset of labour. As multiples pregnancies and iatrogenic births are associated with a number of other complications that could affect gestational age at birth, they were not included in the analysis.

Month of birth may be used to investigate factors directly influencing delivery, and was therefore used to investigate monthly variation in delivery >41 weeks of gestation. Deliveries in the same month, however, have a large difference in time of conception and consequently exposure to risk factors [28]. This study, therefore, primarily focuses on the month of conception. Moreover, since weather in the Netherlands may exhibit large differences throughout a season [33], analyses were done in months rather than seasons. By focusing on the month of conception, we thus use a relevant timescale and allow clinicians to incorporate the result of this work in their pre-conceptional counseling.

GA was based on ultrasound dating or date of assisted reproduction. Conception was assumed at GA of 2 + 0 weeks, and PTB was defined as live birth before GA of 37 + 0 weeks (259 days). Average outdoor temperature, relative humidity and hours of sunshine were calculated for seven-day periods from 20 weeks before delivery up to the day before delivery. These meteorological values were recorded and reported by the Royal Dutch Meteorological Institute at the weather station in de Bilt, Utrecht, i.e., the centrally located and main source for national weather registration [33]. Even though it would be preferred to use data from meteorological stations close to the place of residence for each woman, the place of residence was not available due to anonymized nature of the registry. The meteorological station at De Bilt, located centrally in the Netherlands, is considered representative of the Netherlands. Compared to meteorological data measured in the north and south of the Netherlands, an average error of 1.0 degrees Celsius, 1.8 hours of sunshine and 5.5% relative humidity was found within our study period [33].

### Covariates

Covariates related to preterm birth, month of birth and conception month were considered, including demographic characteristics, reproductive characteristics, health status, lifestyle habits during pregnancy and child characteristics. Demographic characteristics included age (<25 years, 25–29 years, 30–34 years, 35–39 years and ≥40 years), ethnicity (Caucasian, non-Caucasian) and social economic status (SES, SES score). Reproductive characteristics included first gestation (yes/no), primipara (yes/no), multiple pregnancy (yes/no) and induction of labour (yes/no). Health status includes Body Mass Index (BMI) (kg/m2) and maternal hypertension ((yes/no), pregnancy-related hypertension (yes/no)). Lifestyle habits during pregnancy include smoking (no smoking, smoking). Child characteristics include birthweight (grams and percentiles), sex (male, female) and congenital disorder(s) (yes/no).

### Statistical analyses

We included all eligible women, and described the distributions of conception month in participants with different demographic characteristics, reproductive characteristics, health status, living habits during pregnancy and child characteristics. For inter-group comparison, the One-way ANOVA was used for normally continuous data, the Kruskal Wallis test was used for non-normally continuous data and the χ2 test for categorical data.

Cox proportional hazards models were used to analyze the effects of month of conception and the climatic factors on GA at delivery. In these analyses, GA was used as the time axis and preterm birth as outcome to study the effects of month of conception and exposure to temperature (differences), humidity and exposure to light on preterm birth among the stages of pregnancy. We adjusted for ethnicity, primipara, multiple pregnancy, maternal age, SES, smoking, pregnancy-related hypertension and sex of the child. Hazard ratios (HRs) and 95% confidence intervals (CIs) were calculated for month of conception, temperature, difference in average temperature between two consecutive weeks, humidity and hours of sunshine individually. Additionally, a Cox proportional hazards model was applied on all climatic factors combined. These models with combined and individual climatic factors were applied to account for multicollinearity and effect modulation. Due to the nature of the dataset and its selection, the survival times of individuals are independent, and outliers in the data could not occur. Linearity between the predictor variable and log-hazard were checked after the initial model fitting. Potential non-linear effects were assessed by fitting Cox proportional hazards models with and without the interaction between time and the covariate. Goodness of fit was assessed by evaluating Akaike’s information criterion [36]. All statistical analyses were performed using SPSS (version 28, IBM USA). Two-sided p values of less than 0.05 were considered statistically significant.

### Missing data

There were few missing data, with the exception of BMI, for which 89% of data were missing. Although BMI is regarded as a possible factor associated with PTB, including BMI in the analyses would limit the analysis to only 11% of the total data, and BMI was therefore excluded from further analyses.

## Results

### Baseline characteristics

Between 2015 and 2019, 988,787 deliveries were registered by Perined and reviewed by the authors. Of these births, 160,213 were excluded due to missing delivery date or delivery at a GA of less than 24 weeks, after which 828,574 remained (83.8%). Table 1 presents the characteristics of this population. The majority of women were between the age of 25 and 34 (69.2%), and Caucasian of ethnicity (79.0%). Smoking (2.0%), maternal hypertension (0.7%), and multiple pregnancy (3.1%) all had low prevalence in the data. 51.2% of children were male, and 65.5% of births occurred after spontaneous onset. Seasonality in conceptions showed a peak in December and a nadir in February, whereas seasonality in births was found with a peak in July to September and a nadir in February.

**Table 1:** Baseline characteristics.

Characteristics	Total (n = 828574)	Month of conception												Significance
		January (n = 72670)	February (n = 62400)	March (n = 66213)	April (n = 65234)	May (n = 68810)	June (n = 64484)	July (n = 67863)	August (n = 69758)	September (n = 68132)	October (n = 73994)	November (n = 72606)	December (n = 76410)	
**Demographic characteristics**														
**Age (years)**														a/ p = .000^*^
<25	75639	9.0% (6545)	9.3% (5792)	9.6% (6225)	9.6% (6240)	9.5% (6553)	9.4% (6031)	9.1% (6156)	9.2% (6408)	8.7% (5938)	8.8% (6516)	8.8% (6356)	9.0% (6879)	
25-29	250362	30.3% (22021)	29.7% (18558)	30.1% (19957)	30.1% (19606)	30.4% (20940)	30.3% (19561)	30.3% (20541)	30.2% (21060)	30.1% (20538)	30.1% (22282)	30.4% (22069)	30.4% (23229)	
30-34	322740	39.1% (28373)	38.8% (24234)	38.1% (25239)	38.1% (24820)	38.3% (26375)	38.8% (25019)	39.1% (26547)	39.3% (27382)	39.8% (27099)	39.6% (29285)	39.2% (28482)	39.1% (29885)	
35-39	151375	18.2% (13210)	18.6% (11593)	18.7% (12355)	18.7% (12191)	18.3% (12572)	17.9% (11571)	18.2% (12341)	18.1% (12604)	18.1% (12343)	18.1% (13411)	18.2% (13233)	18.3% (13951)	
≥40	28271	3.4% (2499)	3.5% (2211)	3.6% (2418)	3.6% (2354)	3.4% (2358)	3.6% (2291)	3.3% (2261)	3.3% (2289)	3.2% (2205)	3.4% (2485)	3.4% (2448)	3.2% (2452)	
Missing	187	0.0% (22)	0.0% (12)	0.0% (19)	0.0% (23)	0.0% (12)	0.0% (11)	0.0% (17)	0.0% (15)	0.0% (9)	0.0% (15)	0.0% (18)	0.0% (14)	
**Ethnicity**														a/ p = .000^*^
Caucasian	654258	79.2% (57575)	78.6% (49050)	77.8% (51526)	77.9% (50787)	78.6% (54071)	79.7% (51415)	79.2% (53771)	79.0% (55078)	79.1% (53882)	79.4% (58778)	79.6% (57994)	79.2% (60507)	
Non-Caucasian	160715	19.2% (13944)	19.8% (12343)	20.5% (13568)	20.1% (13281)	19.8% (13591)	18.6% (12018)	19.1% (12965)	19.4% (13513)	19.3% (13151)	19.0% (14031)	18.8% (13623)	19.2% (14687)	
Missing	13601	1.6% (1151)	1.6% (1007)	1.7% (1119)	1.8% (1166)	1.7% (1148)	1.6% (1051)	1.7% (1127)	1.7% (1167)	1.6% (1099(	1.6% (1185)	1.6% (1165)	1.6% (1216)	
**SES**	828574	-0.037	-0.074	-0.071	-0.079	-0.036	-0.030	-0.043	-0.028	-0.048	-0.043	-0.037	-0.051	c/ p = .000^*^
**Reproductive characteristics**														
**First gestation**	293179	35.6% (25890)	36.2% (22611)	36.4% (24105)	35.9% (23440)	35.9% (24671)	34.7% (22387)	34.5% (23412)	34.2% (23863)	34.6% (23559)	34.9% (25829)	35.8% (25974)	35.9% (27438)	a/ p = .000^*^
**Primipara**	366383	44.3% (32197)	45.3% (28291)	46.1% (30506)	45.5% (29681)	44.7% (30788)	43.8% (28262)	43.2% (29325)	42.6% (29699)	43.1% (29375)	43.3% (32017)	44.3% (32164)	44.6% (34078)	a/ p = .000^*^
**Multiple pregnancy**	25385	3.2% (2302)	3.3% (2082)	3.1% (2065)	3.2% (2056)	3.0% (2076)	2.9% (1864)	2.9% (1962)	2.9% (2029)	3.1% (2086)	3.1% (2267)	3.2% (2291)	3.0% (2305)	a/ p = .000^*^
**Induction of labour**	258433	32.0% (22534)	32.7% (19722)	33.4% (21317)	32.5% (20439)	32.4% (21534)	32.0% (19945)	32.4% (21234)	31.4% (21169)	31.9% (21054)	32.2% (23065)	32.2% (22720)	32.0% (23700)	a/ p = .000^*^
**Health status**														
**BMI (kg/m2)**	90204	25.0	25.0	25.1	24.9	25.0	24.8	24.9	24.8	24.8	24.8	24.9	24.9	c/ p = .270
Missing	738370	n = 64905	n = 55461	n = 58715	n = 58484	n = 61390	n = 57647	n = 60383	n = 62088	n = 60646	n = 65923	n = 65029	n = 68523	
**Maternal hypertension**	5689	0.7% (522)	0.8% (469)	0.8% (512)	0.8% (487)	0.7% (491)	0.7% (469)	0.7% (488)	0.7% (479)	0.7% (446)	0.6% (447)	0.6% (415)	0.6% (464)	a/ p = .000^*^
Pregnancy related **hypertension**	41165	4.9% (3570)	5.1% (3174)	5.3% (3520)	5.2% (3405)	5.3% (3664)	5.2% (3344)	5.3% (3623)	4.9% (3436)	4.6% (3146)	4.7% (3489)	4.6% (3334)	4.5% (3460)	a/ p = .000^*^
**Living habits during pregnancy**														
**Smoking**														a/ p = .001^*^
No smoking	812203	98.0% (71227)	98.1% (61189)	97.8% (64767)	97.9% (63866)	98.0% (67448)	98.0% (63189)	98.0% (66512)	98.1% (68466)	98.1% (66810)	98.1% (72580)	98.1% (71248)	98.0% (74901)	
Smoking	16371	2.0% (1443)	1.9% (1211)	2.2% (1446)	2.1% (1368)	2.0% (1362)	2.0% (1295)	2.0% (1351)	1.9% (1292)	1.9% (1322)	1.9% (1414)	1.9% (1358)	2.0% (1509)	
**Child characteristics**														
**Birthweight (grams)**	827256	3342	3338	3325	3334	3347	3348	3357	3352	3351	3365	3345	3357	b/ p = .000^*^
**Birthweight (percentiles)**	821685	48	47	47	47	48	48	48	48	48	48	48	48	**c**/ p = .000^*^
**Sex**														a/ p = .123
Male	423997	50.8% (36922)	51.5% (32114)	51.5% (34069)	50.9% (33187)	51.1% (35178)	51.3% (33092)	51.0% (34618)	51.3% (35761)	51.0% (34736)	51.3% (37991)	51.2% (37189)	51.2% (39140)	
Female	403006	49.0% (35623)	48.3% (30143)	48.3% (31998)	48.9% (31915)	48.7% (33503)	48.5% (31287)	48.8% (33116)	48.5% (33860)	48.8% (33274)	48.5% (35860)	48.6% (35298)	48.6% (37129)	
Inconclusive	1098	0.1% (84)	0.2% (100)	0.1% (94)	0.1% (92)	0.1% (100)	0.1% (70)	0.1% (98)	0.1% (102)	0.1% (91)	0.1% (85)	0.1% (82)	0.1% (100)	
Missing	473	0.1% (41)	0.1% (43)	0.1% (52)	0.1% (40)	0.0% (29)	0.1% (35)	0.0% (31)	0.1% (35)	0.0% (31)	0.1% (58)	0.1% (37)	0.1% (41)	
**Congenital disorder**	14971	1.8% (1304)	1.7% (1079)	1.8% (66213)	1.8% (1192)	1.9% (1306)	1.8% (1183)	1.8% (1233)	1.9% (1294)	1.8% (1246)	1.8% (1301)	1.8% (1293)	1.7% (1330)	a/ p = .505

a/ Chi-square test, for categorical data

b/ One way ANOVA, for normally continuous data of the groups

c/ Kruskal Wallis test, for non-normally continuous data of the groups

* Statistical significance (p < 0.05)

### (Preterm) birth seasonality

**Fig 1:**
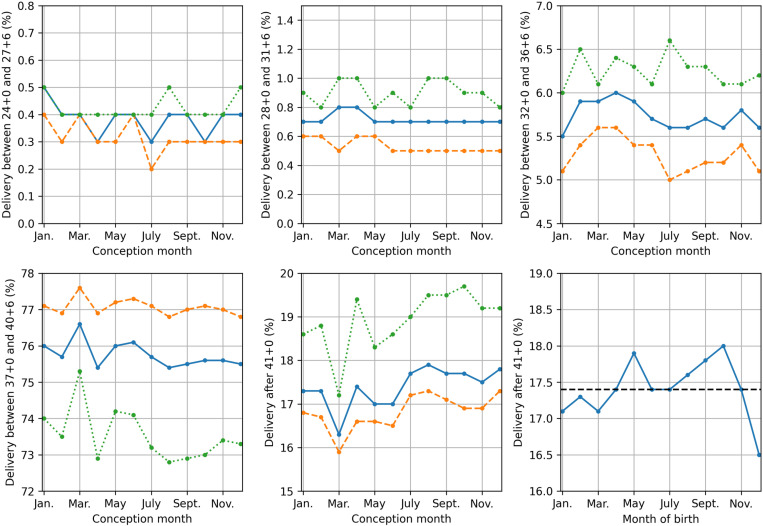
Division of gestational age at birth per conception month showing all births (blue), spontaneous onset of birth (dashed orange) and iatrogenic onset of birth (dotted green) for delivery between 24-28 weeks of gestation (top left), 28-32 weeks of gestation (top middle), 32-37 weeks of gestation (top right), 37-41 weeks of gestation (bottom left) and delivery after 41 weeks of gestation (bottom middle). The bottom right graph shows the percentage of births after 41 weeks of gestation relative to all births in each month, along with the overall percentage of births occurring after 41 weeks of gestation (dashed black).

The distribution of gestational age at birth per conception month (Fig 1) showed significant variation between months when all births, only spontaneous onset of delivery, or only iatrogenic onset of delivery were considered (all p < 0.001).

Preterm and term births were more common in pregnancies conceived between February and June, whereas birth after 41 weeks of gestation was most common in pregnancies conceived between July and December (Fig 1). The percentage of deliveries >41 weeks of gestation, per month of birth, showed significant variation as well (p < 0.001), with a distinct minimum of 16.5% in December and two local maxima at 17.9% and 18.0% for May and October, respectively (Fig 1).

**Fig 2:**
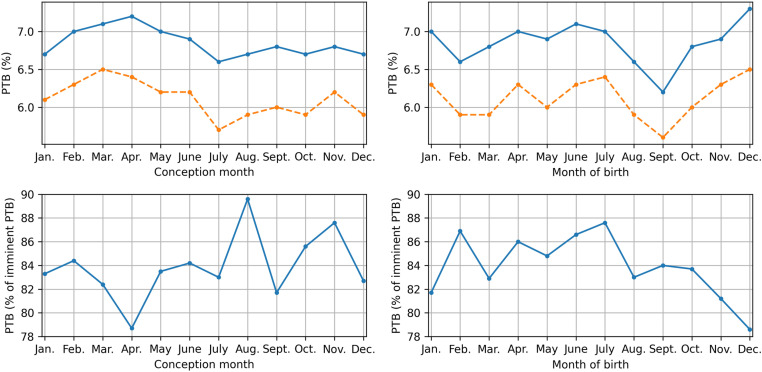
The division of preterm birth (GA < 37 + 0 at time of delivery) for conception month (top left) and month of birth (top right), divided in all deliveries (blue) and spontaneous deliveries (orange dashed). For women diagnosed with imminent preterm birth, the percentage of preterm deliveries is shown by conception month (bottom left) and month of birth (bottom right).

The fraction of PTB in all deliveries was found to differ significantly between the months of conception and birth when all or only spontaneous onset of delivery were considered (all p < 0.001). PTB, both spontaneous and iatrogenic, is most prevalent in pregnancies conceived between February and June and shows a prominent peak for birth in December. A distinct reduction in prevalence of PTB was found in September (Fig 2). The preterm birth rate among women diagnosed with imminent preterm birth did not differ significantly between either month of conception (p = 0.103) or month of birth (p = 0.206). Although the results were not significant, the prevalence of PTB among women diagnosed with imminent preterm birth showed seasonal trends, with an increase for pregnancies conceived in August, October and November or birth occurring in February until July (Fig 2).

**Fig 3:**
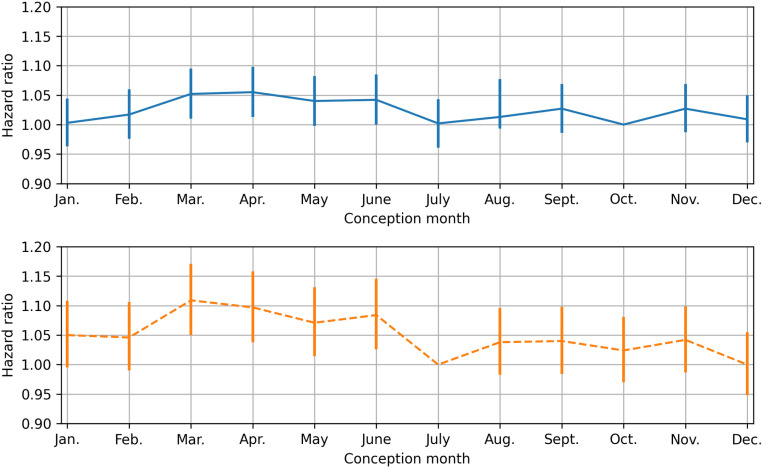
Hazard ratio for preterm birth per conception month for all deliveries (top) and spontaneous births (bottom), adjusted for demographic characteristics (age, ethnicity, SES), reproductive characteristics (first gestation, primipara and multiple pregnancy), health status (pregnancy related hypertension), living habits during pregnancy (smoking) and child characteristics (sex).

The hazard ratio for PTB per month of conception does not vary significantly when all deliveries are considered (p = 0.079), but does so when only spontaneous onset of delivery is used (p = 0.001). For the latter group, the hazard ratio’s for January through June exceed all hazard ratio’s for July through December (Fig 3, S2 Appendix).

Other contributors to PTB were found to be maternal age with HR = 0.955 (95% CI: 0.919–0.992) for age between 25 and 29, HR = 0.919 (95% CI: 0.884–0.955) for age 30–34, HR = 0.966 (95% CI: 0.924–1.009) for age 35–39 and HR = 1.165 (95% CI: 1.087–1.250) for age ≥ 40 years relative to maternal age < 25 years old, ethnicity with HR = 1.045 (95% CI: 1.016–1.075) for non-Caucasian, gravidity with HR = 0.944 (95% CI: 0.910–0.980) for multigravida, parity with HR = 0.629 (95% CI: 0.606–0.653) for multipara, pregnancy-related hypertension with HR = 1.714 (95% CI: 1.604–1.831), smoking HR = 1.621 (95% CI: 1.514–1.735), SES with HR = 0.951 (95% CI: 0.943–0.960), fetal sex with HR = 0.804 (95% CI: 0.787–0.822) for female, and multiple gestation with HR = 32.396 (95% CI: 31.495–33.323).

### Climatic variables and preterm birth

**Fig 4:**
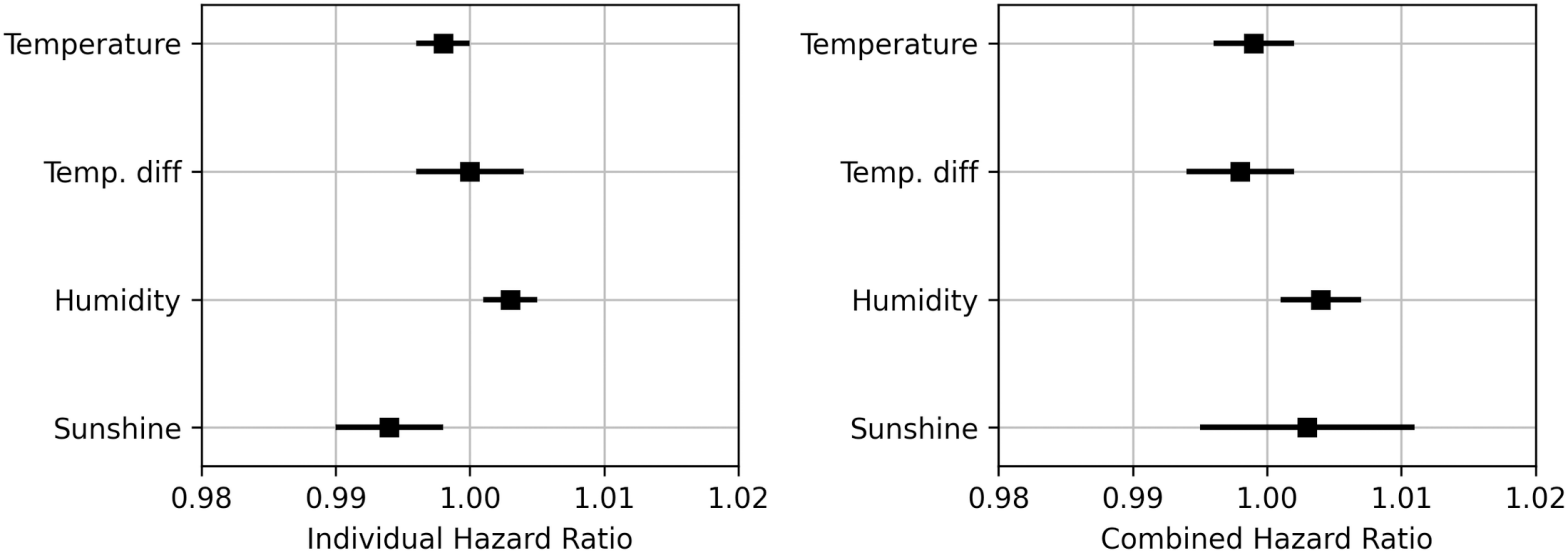
Hazard ratio for the climatic factors on spontaneous onset of preterm birth when analyzed individually (left) and when analyzed as combined inputs for a model (right).

When considering the climatic factors separately, temperature (p = 0.022) with HR = 0.998 (95% CI: 0.996–1.000) and hours of sunshine (p = 0.005) with HR = 0.994 (95% CI: 0.994–0.998) were found to decrease the risk of spontaneous PTB, whereas humidity (p < 0.001) with HR = 1.003 (95% CI: 1.001–1.005) increases the risk of spontaneous PTB. No significant effect for the difference in temperature (p = 0.989) was found. When a combination of these factors was considered, humidity was identified as increasing the risk of spontaneous PTB (p = 0.015) with HR = 1.004 (95% CI: 1.001–1.007), whereas temperature (p = 0.436), difference in temperature (p = 0.352) and hours of sunshine (p = 0.497) were not identified as significant risk factors (Fig 4, S2 Appendix).

## Discussion

In our study, the incidence of preterm birth in the Netherlands was found to exhibit significant variability between months of conception. Deliveries between 32 and 37 weeks of gestation contribute most to preterm birth and its monthly variation. Pregnancies conceived between January through June are associated with a higher risk of preterm birth than those conceived between July and December. The preterm birth rate among women diagnosed with imminent preterm birth was not found to show significant monthly variation. Delivery >41 weeks of gestation showed significant variation over the month of birth, with a minimum in December. An increased risk of preterm birth was associated with decreased ambient temperature, increased ambient humidity and decreased hours of sunlight.

The results found in our work may be a result of seasonal hormonal changes. When considering month of birth, December shows a peak in PTB rate (similar to the UK [23]), along with a Nadir in deliveries >41 weeks of gestation. Additionally, December shows a decrease in the ratio of preterm deliveries after the diagnosis imminent preterm birth (i.e., more false-positive diagnoses, which together with the increased PTB rate, strongly increase the total imminent PTB diagnoses). All three findings may be a result of increased uterine activity governed by heightened melatonin levels during December, and explain the increase in risk of PTB found for conceptions between March and June. Melatonin’s role in the risk of PTB is further endorsed by the significant reduction in risk of PTB for increased light exposure. This reduction may be explained by a reduced risk of vitamin D deficiency, as low vitamin D levels during pregnancy can increase the risk of PTB by causing immune dysregulation, placental dysfunction, increased infection susceptibility, and hormonal imbalances [37,38] Nevertheless, pregnant women in the Netherlands are advised to take vitamin supplementation during pregnancy, making melatonin a more likely explanation for sunshine’s decreasing effect on the risk of PTB. Apart from the peak in preterm birth found in December, a moderate summer increase in preterm birth may be observed (Fig 2). Since this increase is not as pronounced in the spontaneous onset of PTB, this summer increase is mostly driven by iatrogenic preterm births. This suggests a higher incidence of obstetric complications (excluding spontaneous preterm birth) during the summer months. Even though the nature of these iatrogenic births cannot be investigated on the current dataset, this may be a result of raised temperatures, since higher ambient temperatures were shown to increase the risk of obstetric complications [8]. However, since the Dutch population typically has access to artificial cooling, the summer increase in iatrogenic PTB may also be a result of lower melatonin concentrations, as melatonin protects both the fetus and placenta against oxidative stress[13,15]. In August, September, and October, a reduction in spontaneous PTB was found, with a distinct minimum in September (Fig. 2). This coincides with an increasing trend in deliveries >41 weeks of gestation for these months, with a peak in October (Fig. 1). As these months exhibit a reduction in temperature which exceeds the decrease in hours of sunlight [33], these trends in gestational age at birth may be caused by an increase in progesterone [17], in lieu of an increase in melatonin [16]. This interplay between melatonin and progesterone might further explain the increased spontaneous PTB rates during November, December, and January, as artificial heating might halt the increase in progesterone normally associated with increased melatonin the winter months.

Over the past decades, birth seasonality has been widely studied [39–41], presenting various outcomes of peaks and nadirs over the seasons among different countries. Related studies, however, do not distinguish between gestational age at birth (e.g., preterm birth or birth >41 weeks of gestation), thereby raising the question whether the observed birth seasonality is a result of seasonal variation in conceptions or in length of gestation. Our study addresses this caveat by presenting results in both the month of conception and month of birth, and our findings suggest that birth seasonality may not solely reflect seasonal variations in conception, but is influenced by variations in gestational age at birth as well.

Contrary to results found in China [31] and Australia [32], our work found higher temperatures to lower the risk of spontaneous PTB for singleton pregnancies. Moreover, a positive correlation between relative humidity and risk of PTB was found in this work, which was reported by Woo et al. as well [26]. While both outdoor temperature and relative humidity influence temperature perception, the generally low temperature and high humidity in the Netherlands [33] would make humidity the larger influence on thermoregulation. Since the Dutch population generally has access to artificial heating and cooling, we expect the outdoors temperature to be of limited influence on temperature perception. Moreover, the Netherlands has a moderate maritime climate, which limits the exposure of our population to heat stress. In China and Australia, however, large differences in climates may expose part of the population to seasonal heat stress, which may lead to preterm birth [31,42] and thereby skews the relation between ambient temperature and preterm birth. Furthermore, variations in population characteristics may also contribute to the differing results. The Netherlands has a well-developed healthcare system with universal access to prenatal care, which may mitigate some environmental stressors. Additionally, lifestyle factors such as housing conditions and commuting habits may influence exposure to environmental factors differently than in China and Australia. The physiological response to environmental conditions may also differ based on genetic, dietary, and behavioral factors specific to each population [39]. Differences in statistical approaches (e.g., statistical analysis, inclusion of iatrogenic PTB) could also explain the observed discrepancies.

Worldwide studies on the seasonality of preterm birth, investigating the influence of month of conception and month of birth on PTB [18–28], may also have been influenced by the contributing variables stated above. Our work shows a similar pattern to research from Japan [26] and the US [27], where conception in the first half of the year is associated with higher PTB risk. However, findings from China [28] show a different trend, with increased risk for conceptions in winter months. Our findings of PTB per birth month aligns with patterns found in the US [20] and Gambia [21], showing a peak in late summer/early autumn and a nadir in winter. This contrasts with studies in Korea [26], Japan [25], and the UK [23], which indicate higher preterm birth risks in winter months. For a complete oversight of seasonal peaks and nadirs in PTB per conception month and per birth month, see S1 Appendix.

Even though the rate of preterm deliveries after the diagnosis imminent preterm birth was not found to differ significantly between months, a trend towards lower diagnosis specificity in the winter is visible, suggesting overall more uterine activity ultimately not leading to preterm labour. Since studies show the Dutch healthcare system to exhibit a diagnosis specificity of 64% [43] for imminent PTB, the > 78% specificity found in this research suggests the diagnosis of imminent PTB is most likely subject to under-reporting, likely distorting these results.

This study used a large sample size over several years (excluding those affected by the COVID-19 pandemic) and included all eligible births in the Netherlands, preventing in- or exclusion bias. By using month of conception, we can account for differences in gestational age during delivery, and the results found in this research may be used to inform women with high risk of preterm birth about their optimal time of conception. Moreover, to the best of our knowledge, this is the first study investigating sunshine exposure as a risk factor of PTB on a large cohort.

As a limitation of this study, several factors which influence PTB could not be investigated due to a lack of reporting. These include previous PTB, pre-existing diseases, BMI, seasonal infection parameters, season-related living habits, religious affections (e.g., Muslims fasting during the Ramadan [28]), social customs (time of holidays, seasonal marriage), and economic and cultural factors [26]. The factors that were considered, all showed significant influence on PTB, with some having an effect stronger than that of the month of conception. These covariates, however, are known risk factors for PTB and our correction for them further strengthens the results found in this research. The reported effects of temperature and light exposure are expected to be influenced by artificial heating, cooling and lighting, which dampens their effect on the Dutch population.

Moreover, we were not able to account for obstetric management strategies (e.g., progesterone prophylaxis and cervical cerclage) proven to prolong pregnancy for women at risk for PTB, for instance those who had a previous PTB [7]. Protocols for obstetric management may vary between hospitals and years.

Lastly, the combination of several years might have induced biases, as lifestyle trends (e.g., the reduction in smoking) or administrative trends (e.g., improvement in quality of registration) may have influenced our results.

## Conclusion

Pregnancies conceived in the Netherlands between January through June are associated with a higher risk of preterm birth than those conceived between July and December. Preterm birth shows a distinct peak in occurrence in December. There is no significant monthly variation in the preterm birth rate among women diagnosed with imminent preterm birth. Delivery >41 weeks of gestation exhibits significant variation over the month of birth, and is minimum in December. Ambient temperature and hours of sunlight were found to correlate negatively with the risk of preterm birth, while ambient relative humidity was found to correlate positively. These findings may be explained by increased uterine activity in the winter months, which may be a result of increased melatonin levels.

## Supporting information

S1 Appendix Seasonal peaks and nadirs in preterm birth per conception month and per birth month. (XLSX)

S2 Appendix Model outputs for the Cox proportional hazard analyses. (DOCX)
