# Supplementary material for: Seasonal pattern of preterm birth in the Netherlands: A population-based retrospective cohort study
Source: PLoS One. 2025 May 27;20(5):e0324873. doi: 10.1371/journal.pone.0324873 (PMC12111638; doi:10.1371/journal.pone.0324873)
Supplement: S2 Appendix — (DOCX) [file pone.0324873.s002.docx]

**S2 Appendix. Model outputs for the Cox proportional hazard analyses**

1. Preterm birth per conception month for all deliveries

|  | B | SE | Wald | df | Sig. | Exp(B) | 95,0% CI for Exp(B) | |
| --- | --- | --- | --- | --- | --- | --- | --- | --- |
|  |  |  |  |  |  |  | Lower | Upper |
| October |  |  | 18,126 | 11 | ,079 |  |  |  |
| January | ,017 | ,021 | ,640 | 1 | ,424 | 1,017 | ,976 | 1,060 |
| February | ,050 | ,021 | 5,903 | 1 | ,015 | 1,052 | 1,010 | 1,095 |
| March | ,053 | ,021 | 6,580 | 1 | ,010 | 1,055 | 1,013 | 1,098 |
| April | ,039 | ,021 | 3,543 | 1 | ,060 | 1,040 | ,998 | 1,082 |
| May | ,041 | ,021 | 3,750 | 1 | ,053 | 1,042 | 1,000 | 1,085 |
| June | ,002 | ,021 | ,005 | 1 | ,942 | 1,002 | ,961 | 1,043 |
| July | ,033 | ,021 | 2,595 | 1 | ,107 | 1,034 | ,993 | 1,077 |
| August | ,026 | ,021 | 1,607 | 1 | ,205 | 1,027 | ,986 | 1,069 |
| September | ,003 | ,021 | ,017 | 1 | ,897 | 1,003 | ,963 | 1,044 |
| November | ,026 | ,020 | 1,678 | 1 | ,195 | 1,027 | ,987 | 1,069 |
| December | ,009 | ,020 | ,197 | 1 | ,657 | 1,009 | ,970 | 1,050 |
| Maternal age < 25 |  |  | 103,386 | 4 | ,000 |  |  |  |
| Maternal age 25-29 | -,057 | ,016 | 13,582 | 1 | ,000 | ,944 | ,916 | ,973 |
| Maternal age 30-34 | -,087 | ,016 | 31,333 | 1 | ,000 | ,916 | ,889 | ,945 |
| Maternal age 35-39 | -,050 | ,017 | 8,233 | 1 | ,004 | ,951 | ,919 | ,984 |
| Maternal age > 39 | ,114 | ,025 | 20,590 | 1 | ,000 | 1,121 | 1,067 | 1,177 |
| Ethnicity | ,088 | ,011 | 65,951 | 1 | ,000 | 1,092 | 1,069 | 1,115 |
| Gravidity | -,053 | ,015 | 13,396 | 1 | ,000 | ,948 | ,921 | ,976 |
| Parity | -,392 | ,015 | 717,851 | 1 | ,000 | ,676 | ,657 | ,696 |
| PRH | ,805 | ,014 | 3261,903 | 1 | ,000 | 2,236 | 2,175 | 2,299 |
| Smoking | ,352 | ,027 | 167,996 | 1 | ,000 | 1,422 | 1,348 | 1,499 |
| SES | -,041 | ,004 | 127,376 | 1 | ,000 | ,960 | ,953 | ,967 |
| Male child |  |  | 279,213 | 2 | ,000 |  |  |  |
| Female child | -,143 | ,009 | 278,242 | 1 | ,000 | ,867 | ,852 | ,882 |
| Non-conclusive sex | -,200 | ,138 | 2,124 | 1 | ,145 | ,818 | ,625 | 1,072 |
| Mult. Gestation | 2,669 | ,010 | 70380,802 | 1 | ,000 | 14,422 | 14,141 | 14,710 |

2. Preterm birth per conception month for spontaneous births only

|  | B | SE | Wald | df | Sig. | Exp(B) | 95,0% CI for Exp(B) | |
| --- | --- | --- | --- | --- | --- | --- | --- | --- |
|  |  |  |  |  |  |  | Lower | Upper |
| July |  |  | 32,499 | 11 | ,001 |  |  |  |
| January | ,045 | ,028 | 2,569 | 1 | ,109 | 1,046 | ,990 | 1,106 |
| February | ,103 | ,028 | 13,878 | 1 | ,000 | 1,109 | 1,050 | 1,171 |
| March | ,092 | ,028 | 10,984 | 1 | ,001 | 1,097 | 1,038 | 1,158 |
| April | ,069 | ,028 | 6,136 | 1 | ,013 | 1,071 | 1,014 | 1,131 |
| May | ,081 | ,028 | 8,321 | 1 | ,004 | 1,084 | 1,026 | 1,146 |
| June | ,049 | ,027 | 3,123 | 1 | ,077 | 1,050 | ,995 | 1,108 |
| August | ,037 | ,028 | 1,778 | 1 | ,182 | 1,038 | ,983 | 1,096 |
| September | ,039 | ,028 | 1,946 | 1 | ,163 | 1,040 | ,984 | 1,098 |
| October | ,024 | ,027 | ,737 | 1 | ,391 | 1,024 | ,970 | 1,081 |
| November | ,041 | ,027 | 2,246 | 1 | ,134 | 1,042 | ,987 | 1,099 |
| December | ,000 | ,027 | ,000 | 1 | ,998 | 1,000 | ,948 | 1,055 |
| Maternal age < 25 |  |  | 67,641 | 4 | ,000 |  |  |  |
| Maternal age 25-29 | -,046 | ,020 | 5,521 | 1 | ,019 | ,955 | ,919 | ,992 |
| Maternal age 30-34 | -,085 | ,020 | 18,342 | 1 | ,000 | ,919 | ,884 | ,955 |
| Maternal age 35-39 | -,035 | ,023 | 2,388 | 1 | ,122 | ,966 | ,924 | 1,009 |
| Maternal age > 39 | ,153 | ,036 | 18,456 | 1 | ,000 | 1,165 | 1,087 | 1,250 |
| Ethnicity | ,044 | ,014 | 9,496 | 1 | ,002 | 1,045 | 1,016 | 1,075 |
| Gravidity | -,057 | ,019 | 9,188 | 1 | ,002 | ,944 | ,910 | ,980 |
| Parity | -,463 | ,019 | 584,357 | 1 | ,000 | ,629 | ,606 | ,653 |
| PRH | ,539 | ,034 | 253,597 | 1 | ,000 | 1,714 | 1,604 | 1,831 |
| Smoking | ,483 | ,035 | 192,138 | 1 | ,000 | 1,621 | 1,514 | 1,735 |
| SES | -,050 | ,005 | 111,953 | 1 | ,000 | ,951 | ,943 | ,960 |
| Male child |  |  | 380,509 | 2 | ,000 |  |  |  |
| Female child | -,218 | ,011 | 379,951 | 1 | ,000 | ,804 | ,787 | ,822 |
| Non-conclusive sex | -,358 | ,354 | 1,026 | 1 | ,311 | ,699 | ,349 | 1,398 |
| Mult. Gestation | 3,478 | ,014 | 58391,947 | 1 | ,000 | 32,396 | 31,495 | 33,323 |

3. Hazard ratio for singleton preterm birth associated with ambient temperatures

|  | B | SE | Wald | df | Sig. | Exp(B) | 95,0% CI for Exp(B) | | |
| --- | --- | --- | --- | --- | --- | --- | --- | --- | --- |
|  |  |  |  |  |  |  | Lower | Upper |  |
| Ambient temperature | -,002 | ,001 | 5,256 | 1 | ,022 | ,998 | ,996 | 1,000 |  |
| Ethnicity | ,061 | ,016 | 14,773 | 1 | ,000 | 1,062 | 1,030 | 1,096 |  |
| Maternal age < 25 |  |  | 113,189 | 4 | ,000 |  |  |  |  |
| Maternal age 25-29 | -,043 | ,021 | 4,116 | 1 | ,042 | ,958 | ,919 | ,999 |  |
| Maternal age 30-34 | -,085 | ,022 | 15,675 | 1 | ,000 | ,918 | ,880 | ,958 |  |
| Maternal age 35-39 | ,016 | ,025 | ,432 | 1 | ,511 | 1,016 | ,968 | 1,067 |  |
| Maternal age > 39 | ,266 | ,040 | 44,514 | 1 | ,000 | 1,305 | 1,207 | 1,411 |  |
| Gravidity | -,055 | ,021 | 7,008 | 1 | ,008 | ,947 | ,909 | ,986 |  |
| Parity | -,496 | ,021 | 553,471 | 1 | ,000 | ,609 | ,584 | ,635 |  |
| PRH | ,653 | ,037 | 317,262 | 1 | ,000 | 1,921 | 1,788 | 2,064 |  |
| Smoking | ,498 | ,036 | 186,873 | 1 | ,000 | 1,645 | 1,532 | 1,767 |  |
| SES | -,052 | ,005 | 99,356 | 1 | ,000 | ,949 | ,940 | ,959 |  |
| Male child |  |  | 445,062 | 2 | ,000 |  |  |  |  |
| Female child | -,263 | ,012 | 444,321 | 1 | ,000 | ,769 | ,751 | ,788 |  |
| Non-conclusive sex | ,274 | ,447 | ,375 | 1 | ,540 | 1,315 | ,547 | 3,160 |  |

4. Hazard ratio for singleton preterm birth associated with difference in ambient temperature

|  | B | SE | Wald | df | Sig. | Exp(B) | 95,0% CI for Exp(B) | | |
| --- | --- | --- | --- | --- | --- | --- | --- | --- | --- |
|  |  |  |  |  |  |  | Lower | Upper |  |
| Ambient temp. diff. | ,000 | ,002 | ,000 | 1 | ,989 | 1,000 | ,996 | 1,004 |  |
| Ethnicity | ,061 | ,016 | 14,779 | 1 | ,000 | 1,062 | 1,030 | 1,096 |  |
| Maternal age < 25 |  |  | 113,402 | 4 | ,000 |  |  |  |  |
| Maternal age 25-29 | -,044 | ,021 | 4,193 | 1 | ,041 | ,957 | ,918 | ,998 |  |
| Maternal age 30-34 | -,086 | ,022 | 15,847 | 1 | ,000 | ,918 | ,880 | ,957 |  |
| Maternal age 35-39 | ,016 | ,025 | ,414 | 1 | ,520 | 1,016 | ,968 | 1,067 |  |
| Maternal age > 39 | ,266 | ,040 | 44,411 | 1 | ,000 | 1,304 | 1,206 | 1,410 |  |
| Gravidity | -,054 | ,021 | 6,911 | 1 | ,009 | ,947 | ,909 | ,986 |  |
| Parity | -,496 | ,021 | 553,960 | 1 | ,000 | ,609 | ,584 | ,635 |  |
| PRH | ,654 | ,037 | 317,850 | 1 | ,000 | 1,922 | 1,789 | 2,066 |  |
| Smoking | ,498 | ,036 | 186,824 | 1 | ,000 | 1,645 | 1,532 | 1,767 |  |
| SES | -,052 | ,005 | 99,439 | 1 | ,000 | ,949 | ,940 | ,959 |  |
| Male child |  |  | 444,975 | 2 | ,000 |  |  |  |  |
| Female child | -,263 | ,012 | 444,228 | 1 | ,000 | ,769 | ,751 | ,788 |  |
| Non-conclusive sex | ,275 | ,447 | ,379 | 1 | ,538 | 1,317 | ,548 | 3,165 |  |

5. Hazard ratio for singleton preterm birth associated with ambient humidity

|  | B | SE | Wald | df | Sig. | Exp(B) | 95,0% CI for Exp(B) | | |
| --- | --- | --- | --- | --- | --- | --- | --- | --- | --- |
|  |  |  |  |  |  |  | Lower | Upper |  |
| Ambient humidity | ,003 | ,001 | 13,209 | 1 | ,000 | 1,003 | 1,001 | 1,005 |  |
| Ethnicity | ,061 | ,016 | 14,765 | 1 | ,000 | 1,062 | 1,030 | 1,096 |  |
| Maternal age < 25 |  |  | 112,812 | 4 | ,000 |  |  |  |  |
| Maternal age 25-29 | -,043 | ,021 | 4,082 | 1 | ,043 | ,958 | ,919 | ,999 |  |
| Maternal age 30-34 | -,085 | ,022 | 15,594 | 1 | ,000 | ,919 | ,881 | ,958 |  |
| Maternal age 35-39 | ,016 | ,025 | ,433 | 1 | ,511 | 1,016 | ,968 | 1,067 |  |
| Maternal age > 39 | ,266 | ,040 | 44,420 | 1 | ,000 | 1,304 | 1,206 | 1,410 |  |
| Gravidity | -,055 | ,021 | 6,998 | 1 | ,008 | ,947 | ,909 | ,986 |  |
| Parity | -,495 | ,021 | 552,378 | 1 | ,000 | ,609 | ,585 | ,635 |  |
| PRH | ,653 | ,037 | 316,921 | 1 | ,000 | 1,921 | 1,787 | 2,064 |  |
| Smoking | ,497 | ,036 | 186,424 | 1 | ,000 | 1,644 | 1,531 | 1,766 |  |
| SES | -,052 | ,005 | 99,149 | 1 | ,000 | ,949 | ,940 | ,959 |  |
| Male child |  |  | 445,083 | 2 | ,000 |  |  |  |  |
| Female child | -,263 | ,012 | 444,331 | 1 | ,000 | ,769 | ,751 | ,788 |  |
| Non-conclusive sex | ,277 | ,447 | ,383 | 1 | ,536 | 1,319 | ,549 | 3,169 |  |

6. Hazard ratio for singleton preterm birth associated with of hours of sunlight

|  | B | SE | Wald | df | Sig. | Exp(B) | 95,0% CI for Exp(B) | | |
| --- | --- | --- | --- | --- | --- | --- | --- | --- | --- |
|  |  |  |  |  |  |  | Lower | Upper |  |
| Hours of sunshine | -,006 | ,002 | 7,774 | 1 | ,005 | ,994 | ,990 | ,998 |  |
| Ethnicity | ,061 | ,016 | 14,737 | 1 | ,000 | 1,062 | 1,030 | 1,096 |  |
| Maternal age < 25 |  |  | 113,060 | 4 | ,000 |  |  |  |  |
| Maternal age 25-29 | -,043 | ,021 | 4,113 | 1 | ,043 | ,958 | ,919 | ,999 |  |
| Maternal age 30-34 | -,085 | ,022 | 15,674 | 1 | ,000 | ,918 | ,880 | ,958 |  |
| Maternal age 35-39 | ,016 | ,025 | ,427 | 1 | ,513 | 1,016 | ,968 | 1,067 |  |
| Maternal age > 39 | ,266 | ,040 | 44,457 | 1 | ,000 | 1,304 | 1,206 | 1,411 |  |
| Gravidity | -,055 | ,021 | 6,990 | 1 | ,008 | ,947 | ,909 | ,986 |  |
| Parity | -,496 | ,021 | 552,938 | 1 | ,000 | ,609 | ,585 | ,635 |  |
| PRH | ,653 | ,037 | 317,179 | 1 | ,000 | 1,921 | 1,788 | 2,064 |  |
| Smoking | ,498 | ,036 | 186,678 | 1 | ,000 | 1,645 | 1,532 | 1,767 |  |
| SES | -,052 | ,005 | 99,284 | 1 | ,000 | ,949 | ,940 | ,959 |  |
| Male child |  |  | 445,028 | 2 | ,000 |  |  |  |  |
| Female child | -,263 | ,012 | 444,280 | 1 | ,000 | ,769 | ,751 | ,788 |  |
| Non-conclusive sex | ,276 | ,447 | ,380 | 1 | ,538 | 1,317 | ,548 | 3,166 |  |

7. Hazard ratio for singleton preterm birth associated with all four climatic factors

|  | B | SE | Wald | df | Sig. | Exp(B) | 95,0% CI for Exp(B) | | |
| --- | --- | --- | --- | --- | --- | --- | --- | --- | --- |
|  |  |  |  |  |  |  | Lower | Upper |  |
| Ambient temperature | -,001 | ,001 | ,606 | 1 | ,436 | ,999 | ,996 | 1,002 |  |
| Ambient temp. diff. | -,002 | ,002 | ,867 | 1 | ,352 | ,998 | ,994 | 1,002 |  |
| Ambient humidity | ,004 | ,001 | 5,886 | 1 | ,015 | 1,004 | 1,001 | 1,007 |  |
| Hours of sunshine | ,003 | ,004 | ,462 | 1 | ,497 | 1,003 | ,995 | 1,011 |  |
| Ethnicity | ,061 | ,016 | 14,792 | 1 | ,000 | 1,063 | 1,030 | 1,096 |  |
| Maternal age < 25 |  |  | 112,795 | 4 | ,000 |  |  |  |  |
| Maternal age 25-29 | -,043 | ,021 | 4,066 | 1 | ,044 | ,958 | ,919 | ,999 |  |
| Maternal age 30-34 | -,085 | ,022 | 15,554 | 1 | ,000 | ,919 | ,881 | ,958 |  |
| Maternal age 35-39 | ,016 | ,025 | ,439 | 1 | ,508 | 1,017 | ,968 | 1,067 |  |
| Maternal age > 39 | ,266 | ,040 | 44,454 | 1 | ,000 | 1,304 | 1,206 | 1,410 |  |
| Gravidity | -,055 | ,021 | 7,021 | 1 | ,008 | ,947 | ,909 | ,986 |  |
| Parity | -,495 | ,021 | 552,412 | 1 | ,000 | ,609 | ,585 | ,635 |  |
| PRH | ,653 | ,037 | 316,804 | 1 | ,000 | 1,920 | 1,787 | 2,063 |  |
| Smoking | ,497 | ,036 | 186,451 | 1 | ,000 | 1,645 | 1,531 | 1,766 |  |
| SES | -,052 | ,005 | 99,141 | 1 | ,000 | ,949 | ,940 | ,959 |  |
| Male child |  |  | 445,137 | 2 | ,000 |  |  |  |  |
| Female child | -,263 | ,012 | 444,388 | 1 | ,000 | ,769 | ,751 | ,788 |  |
| Non-conclusive sex | ,276 | ,447 | ,381 | 1 | ,537 | 1,318 | ,548 | 3,167 |  |
